# Vaccination for Smallpox

**Published:** 1909-01

**Authors:** James B. Colegrove

**Affiliations:** Washington, D. C.


### Vaccination for Smallpox. Should it be Discontinued and Abolished?

By JAMES B. COLEGROVE, M. D., Washington, D. C.

I HAVE observed that there has been some propositions made in medical societies that vaccination as a preventive for smallpox be discontinued,—that there is more harm done by vaccination than by the disease itself. I have seen several published statements of this character, in which it was strongly urged that vaccination should be abolished. The subject seems to have attracted considerable attention, and I cannot believe that a thoughtful physician would urge this, if the history of smallpox is carefully considered. It is claimed that in some instances blood poisoning has occurred as a result of vaccination, and no doubt this is true. But the number of such cases bears no comparison to the many who have been vaccinated with no untoward results. Erysipelatous inflammation, or suppuration is sometimes, but rarely known to occur, excepting when the person is peculiarly susceptible to skin diseases.

### HISTORY OF SMALLPOX.

Our present knowledge of the first appearance of this dread scourge was given by an Arabic physician named Phazes in the eight century. It first appeared, so far as we know, in southeastern Europe, and it prevailed all over Italy, as well as southern Europe during the ninth century. It invaded France and Spain, and spread with terrible violence through Austria and Hungary, and finally it devastated Prussia and England. In the following century it ravished Northern Europe and spread to Sweden and Norway. There was no known method of treatment that could in the least degree prevent the disease, and the whole of Europe was filled with victims who had survived the attacks of the disease, whose faces bore evidence of the awful results of the dread visitation.

In 1620, it appeared in San Domingo, and thence it traveled to Mexico, where four millions of people died from the fell destroyer. In 1740 it swept over South America, where several millions succumbed to the malady. Again, in the same year, it broke out in Northern Europe, and crossed the cold waters of the north to Iceland and finally to Greenland, which latter country was nearly depopulated.

### HORRIBLE SUFFERING.

No language can adequately describe the horrors, the despair, the woe suffered by the people in Europe in the fifteenth and sixteenth centuries from smallpox. During these dread visitations there was no remedy, no help, nothing that could alleviate the woe-bestricken people. In the years between 1700 and 1770, the scourge swept all over Germany, carrying away millions, and leaving a trail of sorrow and death which no pen can portray. No disease was ever more contagious, and none which rendered its victims more helpless. But, after forty millions of people had been destroyed by this malady Jenner most providentially appeared to the world, and put in check its ravages.

Edward Jenner was born in 1749. He was the son of an obscure vicar in Gloucester, England. He was the one who was destined to rescue the world from the devastation of this terrible pest. His career was both strange and remarkable, and no one imagined that he would rise to imperishable fame and honor. He emerged as it were from oblivion, to a renown few men have reaped in this world. He was first apprenticed to a man named Daniel Ludlow who lived at Sudberry, where he studied for the profession of medicine and surgery. Later he was a student in St. George's Hospital in London. He was then 30 years of age. He was extremely ambitious to reach a high place in the profession. After a voyage for his health, he returned to his native town at Berkley, and began the practice of medicine and surgery. Meantime, after mature study and thought, he began to experiment in vaccination. For two years he followed in this line of investigation, and after having secured virus from a young heifer, he began vaccination of young persons, who consented to be exposed to smallpox. Considerable notoriety arose from his experiments, and he was bitterly attacked in London, by the medical profession and, remarkable to state, also by the clergy. Nevertheless, he persevered and finally announced that smallpox could be prevented. Committees from the profession of London visited him and were confounded by demonstrations of actual cases. Then came the announcement which was made by these committees to the world that Jenner's discovery was a real and true one, and that he was entitled to all honor.

This announcement was like the proclamation of a revolution. Immediately agents from Germany, Austria, France, and Spain were sent to see him and obtain the virus, together with instructions as to the manner of its use. He was overwhelmed with honors. The Parliament voted him $10,000 This he declined, declaring that his reward was in the realisation of what he had accomplished. But so great was the sentiment of gratitude in England, that Parliament a year later, again voted him a donation of $100,000. He was the recipient of other gifts from European nations, and thousands of pounds sterling were laid at his feet from Asiatic countries. The Empress of Russia sent him an autograph letter, accompanied by a diamond of immense value. She notified him that she had vaccinated her first child whom she had named "Vacinnoff."

And now honors came thick and fast to Edward Jenner. His name was presented to institutions of learning all over Europe, in which he was made a fellow or member. The British Government ordered that the army should be vaccinated, and vaccination was made compulsory in Germany, as well as in all the other countries in Europe. Also became the practice in the United States.

### SMALLPOX EXTERMINATED.

So this terror that had swept away its millions was exterminated as a scourge from Europe in a single decade. And in another decade it was driven from the whole world! It would take too much time and space to mention the evidences of gratitude manifested by the nations of Europe towards this remarkable man, Edward Jenner. His name was a household word, and praise was given to Almighty God in the churches in England, for Edward Jenner. A monument was erected to his memory in London during his lifetime, and later, after his death, a statute was placed in the Cathedral at Gloucester, near his birthplace. Among the men who have arisen to lasting fame none has better deserved the gratitude of mankind. Jenner died in 1842. Today, men have not forgotten him. The medical profession know that were vaccination unknown, it would be impossible to stop the plague. No doubt there are remedies that might ameliorate the conditions, but in crowded tenements, and certain localities smallpox wou'd again spread over the world.

### VACCINATION CANNOT BE DISCONTINUED.

It is absurd to insist that vaccination be discontinued. Those who have urged this, have pointed to instances of blood poisoning. These instances, I again declare are isolated, and when compared to the number who have received no injury from vaccination are as one in a thousand—probably a less proportion. It is impossible to always secure absolutely pure virus, but generally, at this time virus is very pure. In fact, there are records of thousands of young persons and children in whom no damage has occurred from vaccination. All over the civilised world, now, it is made compulsory, and very properly so.

Perhaps another Jenner will arise and announce to the world that a prophylactic has been discovered which will prevent tuberculosis, and drive this dread malady from the homes of mankind! Let us so hope and pray!

1000 MARYLAND AVENUE.

